# A Cure for Drunkenness

**Published:** 1888-01

**Authors:** 


**A Cure for Drunkenness.**—A half ounce of ground quassia steeped in a pint of vinegar, is recommended highly as a cure for drunkenness. A teaspoonful in a little water should be taken every time the liquor thirst is felt. It satisfies the cravings and produces a feeling of stimulation and strength.—*Medical World*.

